# A Case Report of Autoimmune Phenomena With Underlying Breast Cancer: Occam’s Razor Versus Hickam’s Dictum

**DOI:** 10.7759/cureus.25511

**Published:** 2022-05-31

**Authors:** Parima Saxena, Hussam R Alkaissi, Harjinder Gill, Samy I. McFarlane

**Affiliations:** 1 Internal Medicine, State University of New York Downstate Medical Center, Brooklyn, USA; 2 Internal Medicine, Kings County Hospital Center, Brooklyn, USA; 3 Internal Medicine, Veterans Affairs Medical Center, Brooklyn, USA; 4 Medicine and Endocrinology, State University of New York Downstate Medical Center, Brooklyn, USA

**Keywords:** hickam’s dictum, occam's razor, breast cancer, pernicious anaemia, evan’s syndrome, immune hemolytic anemia, poly-autoimmunity, autoimmune phenomena

## Abstract

The association between malignancies and autoimmunity had been well-established. The proposed pathophysiology and causality can be bidirectional. For example, a paraneoplastic syndrome can be triggered by an underlying malignancy or vice versa, where chronic inflammation of organs affected by autoimmunity can induce malignant transformation such as the case with inflammatory bowel disease and colorectal cancer or primary sclerosing cholangitis and hepatobiliary cancer. This report presents a case of autoimmune phenomena, namely, autoimmune hemolytic anemia, pernicious anemia, and Graves disease associated with newly diagnosed breast cancer. We also highlight the postulated pathophysiologic mechanisms in an attempt to answer the question of whether the occurrence of these autoimmune phenomena in our patient is a result of the law of parsimony (Occam's razor), where clinical variables are pathogenically related, or the counterargument, where random events and diseases can take place simultaneously (Hickam's dictum).

## Introduction

The association between autoimmune diseases and malignancies had been long established. The chronic inflammatory state may contribute to malignant transformation, such as the case of inflammatory bowel disease and colon cancer, rheumatoid arthritis and systemic lupus erythematous predisposing to B-cell lymphoma, and Sjögren's syndrome, where germinal center inflammation can lead to non-Hodgkin lymphoma [1]. On the other hand, malignancy can also trigger the development of autoimmunity through various paraneoplastic syndromes [2]. This is observed in ovarian cancer with cerebellar degeneration through the production of anti-Yo antibodies [1]. While both processes can lead to the development of the other, it remains unclear which condition is the cause and which is the effect.

This report presents a unique case of multiple new-onset autoimmune phenomena in a patient whose further workup revealed an underlying malignancy.

## Case presentation

A 55-year-old woman presented with new-onset fatigue and dyspnea on exertion for three weeks. She also reported night sweats but denied chest pain, weight loss, or fever. She had a past medical history of hypertension treated with losartan, class 2 obesity with a body mass index of 35, and multinodular goiter. Family history is significant for breast cancer in her mother and sister (post-menopausal in both cases). Her last mammogram was 16 years prior to her presentation and was normal. Her last colonoscopy was five years prior to presentation and was unremarkable. Her menopause started at the age of 50, and she had a history of oral contraceptive use for one year in the distant past.

On initial examination, she was tachycardic up to 108 beats per minute, with normal blood pressure, and she was afebrile. The rest of her physical examination was otherwise unremarkable. Initial blood work showed anemia with hemoglobin of 6.9 g/dl with neutropenia and mild thrombocytopenia (Table 1). Hemolysis workup was positive, with elevated lactate dehydrogenase (LDH) up to 6656 U/L, undetectable haptoglobin, and up-trending bilirubin. Coombs test was positive, establishing the diagnosis of autoimmune hemolytic anemia. The patient was treated with prednisone 80 mg daily (1 mg/kg/day) for five days and received one unit of packed red blood cells. The hemoglobin, white cell, and platelet count up-trended after starting glucocorticoids. LDH and haptoglobin normalized as well. The patient's prior medical records showed a high mean corpuscular volume (MCV) of 112 fl, with mild anemia with hemoglobin of 11.8 g/dl one year prior to her current admission. She also had a low vitamin B12 of 150 pg/mL, with a positive intrinsic factor and parietal cell autoantibodies. Otherwise, antinuclear antibodies were negative and immunoglobulin A (IgA) levels were normal, with no monoclonal gammopathy on serum and urine protein electrophoresis. She had normal iron studies with elevated ferritin. HIV serology was negative.

**Table 1:** Laboratory data at baseline, hospitalization, and following treatment of the breast cancer (over a period of two years) LDH: lactate dehydrogenase

Variable vs. Time	5/2020	3/23/2021	3/24/2021	3/25/2021	3/26/2021	3/27/2021	3/28/2021	3/29/2021	3/30/2021	5/2021	2/2022	3/2022	Reference range
White-cell count (per µl)	3.25	2.8	2.7	2.7	5.3	7.3	9.6	7.3	7.3	4	3.6		3,500-10,800
Hemoglobin (g/dl)	11.8	6.9	6.7	8.5	7.9	7.6	7.6	7.9	8.5	11.8	12		12-16
Hematocrit (%)	33.8	20.2	19.4	24.5	23.3	22.7	22.7	23.3	26.5	99.2	37.8		35-45
Platelets count (1000 per µl)	203	113	121	126	94	83	83	93	127	231	174		150-350
Mean corpuscular volume (fl)	112	115	114	103	106	107	108	110	111	99	87		78-95
Reticulocyte count (absolute, 1000 per µl)		150								46.7			0.09-0.13
Reticulocyte count (percentage)		0.85								1.3			0.5-2.9
Total bilirubin		1.4	2.1	1.7	1.1	0.5	0.5	NA	NA	0.2	0.5		
Haptoglobin (mg/dL)		<10								126			34-200
LDH (U/L)		6,656								312			50-240
Fibrinogen (mg/dL)			261										200-400
Direct antiglobulin			Positive										Negative
Ferritin (ng/mL)			982										50-150
Vitamin B12 (pg/mL)			150							1,900			232-1,245
Anti-parietal cell antibodies (titer)			1:80										<1:20
Intrinsic factor antibodies (AU/mL)			172										<1.1

We further examined the patient for clues for this flare of multiple autoimmune hematological disorders. The patient said she recently noticed a small lump in the right breast confirmed on a breast exam where a small irregular retroareolar mass was palpable.

**Figure 1:**
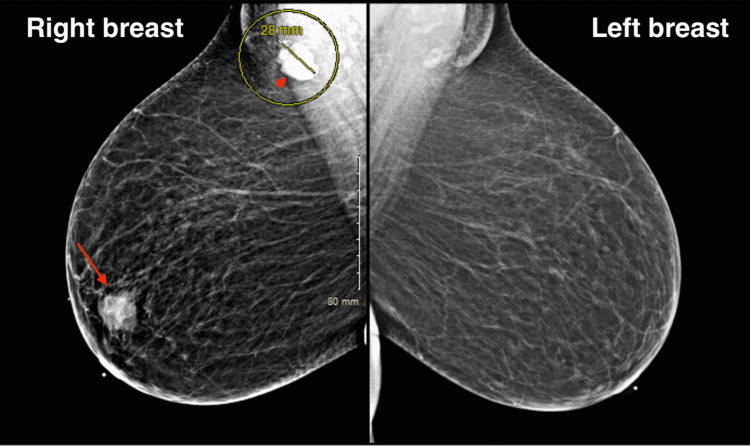
Mammogram of the right and left breasts, showing a retroareolar mass in the right breast (arrow) with an enlarged ipsilateral lymph node (arrowhead)

**Figure 2:**
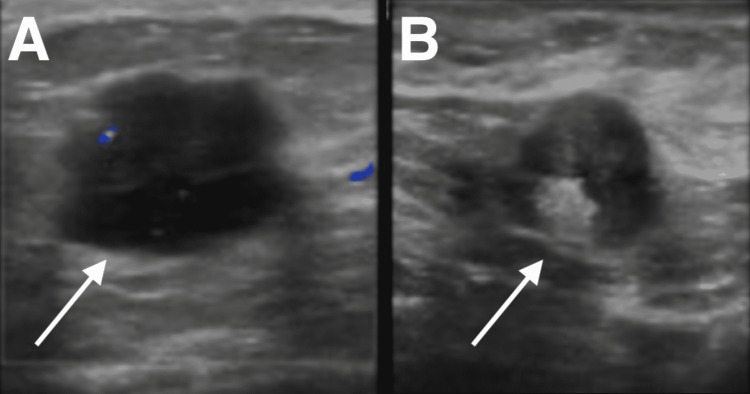
Ultrasound of the breast showing (a) irregularly-shaped, hypodense, retroareolar mass (arrow), (b) enlarged ipsilateral axillary lymph node with lobulated contour, increased width, widest diameter of 2.8 cm, with preserved hilum (arrow)

The mammogram showed two masses and an enlarged axillary lymph node (Figure 1). Ultrasound of the breast showed a retroareolar, irregular-shaped mass of 3 cm at the widest diameter (Figure 2a). In addition, a prominent axillary lymph node with preserved hilum has a lobulated structure, 2.8 cm at the widest diameter (Figure 2b).

Histopathology showed invasive ductal adenocarcinoma, grade 3, from the retroareolar mass, with otherwise normal breast tissue from the other mass. The ipsilateral axillary lymph node showed metastatic breast cancer. Immunohistochemistry stained weakly positive for estrogen receptor (ER) with a percentage of 6.77% and human epidermal growth factor receptor 2 (HER2) with a score of 3+ but negative progesterone receptor (PR) expression. Further genetic analysis showed no evidence of any hereditary predisposing variants. No evidence of skeletal metastases on the bone scan. The patient was diagnosed with breast cancer stage IIB (T2, N1, M0). The patient received six cycles of docetaxel and carboplatin and planned to continue to receive trastuzumab and pertuzumab for one year. Repeated imaging showed no evidence of any mass, so the patient was planned to have breast-conserving surgery. Thus, she underwent a right lumpectomy, and a biopsy of the lymph node bed showed no evidence of any malignant findings.

Ten months later, she started having weight loss, despite having a normal appetite, with no nausea. She lost around 10 lbs over two months. The evaluation showed newly undetectable thyroid-stimulating hormone (TSH), from a baseline of 3.57 µIU/mL that was checked routinely a year prior to presentation (Table 2), with very high titers of thyroid-stimulating immunoglobulin and anti-thyroid peroxidase antibodies. She was diagnosed with Graves disease and started on methimazole 20 mg daily, slowly down titrated over four months to 5 mg a day, establishing clinical and biochemical euthyroid function.

**Table 2:** Thyroid function laboratory data at baseline, after the development of Graves disease (2/2022), and treatment with methimazole (3/2022) TSH: thyroid-stimulating hormone, T4: thyroxine, T3: triiodothyronine

Variable vs Time	5/2021	2/2022	3/2022	Reference range
TSH (µIU/mL)	3.57	<0.01	4.35	0.27-4.2
T4, free (ng/dL)	1.1	2		0.9-1.8
T3 (ng/dL)		194		90-200
Anti-thyroid peroxidase antibodies (IU/mL)		15,263		<35
Thyroid-stimulating immunoglobulin (IU/L)		7.8		<0.55
TSH receptor antibody (IU/L)		5.8		<1.75

## Discussion

Here, we present a unique case of multiple autoimmune diseases in the setting of underlying metastatic breast cancer. Interestingly, in our case, the temporal relation of autoimmunity to cancer is staggered. Our patient presented with symptomatic anemia and was diagnosed with Evans syndrome (autoimmune hemolytic anemia (AIHA) with milder autoimmune neutropenia and immune thrombocytopenia purpura (ITP)), in addition to pernicious anemia, leading to the diagnosis of breast cancer. Several months later, she developed Graves disease after breast cancer treatment. The question is whether this is a case of Occam's razor, "plurality should not be posited without necessity," where both malignancy and autoimmunity are related, or a case of Hickam's dictum, where "patients can have as many diseases as they darn well please” [3], without clear association?

Regarding AIHA, studies have shown that AIHA and Evans syndrome are usually linked to liquid tumors and lymphoproliferative disorders, with limited association with solid tumors [4-5]. However, Kamesaki, in 2016, suggested that AIHA can be a rare paraneoplastic syndrome associated with solid tumors and suggested that tumor antigens may cross-react with erythrocyte antigens, triggering AIHA [6]. One case report by Ugoeke et al. (2017) also identified the presence of AIHA in metastatic breast cancer. The patient presented similarly, where breast cancer was detected in the workup of anemia, which had improved following treatment with chemotherapy. The authors suggested that improvement of anemia symptoms with chemotherapy points towards a paraneoplastic process as the cause of AIHA [7]. Our patient responded appropriately to steroids prior to initiation of docetaxel; however, the temporal association of AIHA to her breast cancer diagnosis points to a paraneoplastic process as the cause of AIHA.

Like AIHA, pernicious anemia has a limited association, with a decreased risk associated with breast cancer [8]. A large-scale study involving 209,000 breast cancer patients and 200,000 healthy controls by Schairer et al. in 2018 studying the association of various autoimmune diseases found reduced breast cancer risk among rheumatoid arthritis (OR = 0.84; 99.9% CI 0.79-0.89), systemic lupus erythematosus (OR = 0.82; 99.9% CI 0.70-0.97), and pernicious anemia patients (OR = 0.90; 99.9% CI 0.83-0.97) and increased risk among those with psoriasis (OR = 1.16; 99.9% CI 1.06-1.27) [8]. A meta-analysis by Lahner et al. in 2018 showed similar findings, with a decreased relative risk of pernicious anemia in breast cancer patients and other solid malignancies but an increased risk with liquid tumors [9]. To date, there have been no reports describing the association between breast cancer and pernicious anemia. Despite this, given the concurrence of multiple autoimmune phenomena, we suggest that pernicious anemia in our patient may have resulted from cross-reactivity between tumor antigens and gastric mucosa as the underlying pathogenetic mechanism of pernicious anemia.

Thyroid autoimmunity and breast cancer have been well-established, primarily with hypothyroidism. Jha et al. conducted a prospective case-control study to determine the rate of thyroid autoimmunity in breast cancer patients. They noted a significantly higher rate of clinical hypothyroidism in breast cancer patients than in the healthy control group [10]. There are limited associations describing breast cancer preceding the onset of Graves disease. Instead, studies have shown that hyperthyroidism moderately increases the relative risk of breast cancer [11]. Chemotherapy also can play a role in thyroid dysfunction. Studies have demonstrated that because of similarities between the breast and thyroid glandular tissue, chemotherapeutic agents may sensitize thyroid tissue to radiotherapy and, therefore, decrease thyroid function. Likewise, tamoxifen and trastuzumab can elicit anti-thyroid effects; however, the mechanism remains unclear. The tissue antigenic similarity and tissue structure of the breast and thyroid may also play a role in breast cancer inducing thyroid dysfunction [12]. After a breast cancer diagnosis, Graves disease onset in our patient can be attributed to different causes. First, surgical intervention, chemotherapy, hormone therapy, and HER2 antagonists may have led to thyroid dysfunction, thus triggering Graves disease. Second, it is also possible that malignant cells in our patient exhibited molecular mimicry to thyroid tissue, thus driving the production of anti-thyroid peroxidase antibodies. However, the temporal association of Graves disease after starting cancer treatment likely is in favor of the first hypothesis, where thyroid tissue damage is likely the inciting factor for Graves disease.

## Conclusions

Given the incidence of multiple autoimmune conditions in our patient, we suggest the possibility of malignant cells expressing self-antigens triggering the development of autoimmune antigens. Overall, our case highlights a unique and unrecognized association of multiple autoimmune phenomena in a patient with underlying breast cancer. More importantly, our case elicits the need to consider underlying malignancy when performing a workup for a patient with a presentation or history of multiple autoimmune events that do not fulfill any of the common autoimmune syndromes. Further studies on this topic may involve prospective studies in breast cancer patients to evaluate the presence of auto-antibodies, autoimmunity development, and basic research on molecular mimicry in breast cancer tissues that may elicit the loss of tolerance and autoimmunity.
